# Cyanidin-3-*O*-β-glucoside regulates fatty acid metabolism via an AMP-activated protein kinase-dependent signaling pathway in human HepG2 cells

**DOI:** 10.1186/1476-511X-11-10

**Published:** 2012-01-13

**Authors:** Honghui Guo, Guoling Liu, Ruimin Zhong, Yun Wang, Duan Wang, Min Xia

**Affiliations:** 1 Department of Food Science, Yingdong College of Bioengineering, Shaoguan University, Shaoguan, Guangdong Province, China; 2 Guangdong Provincial Key Laboratory of Food, Nutrition and Health; Department of Nutrition, School of Public Health, Sun Yat-sen University (Northern Campus), Guangzhou, Guangdong Province, China

**Keywords:** anthocyanin, AMP-activated protein kinase, acetyl CoA carboxylase, carnitine palmitoyl transferase 1, fatty acid metabolism

## Abstract

**Background:**

Hepatic metabolic derangements are key components in the development of fatty liver disease. AMP-activated protein kinase (AMPK) plays a central role in controlling hepatic lipid metabolism through modulating the downstream acetyl CoA carboxylase (ACC) and carnitine palmitoyl transferase 1 (CPT-1) pathway. In this study, cyanidin-3-*O*-β-glucoside (Cy-3-g), a typical anthocyanin pigment was used to examine its effects on AMPK activation and fatty acid metabolism in human HepG2 hepatocytes.

**Results:**

Anthocyanin Cy-3-g increased cellular AMPK activity in a calmodulin kinase kinase dependent manner. Furthermore, Cy-3-g substantially induced AMPK downstream target ACC phosphorylation and inactivation, and then decreased malonyl CoA contents, leading to stimulation of CPT-1 expression and significant increase of fatty acid oxidation in HepG2 cells. These effects of Cy-3-g are largely abolished by pharmacological and genetic inhibition of AMPK.

**Conclusion:**

This study demonstrates that Cy-3-g regulates hepatic lipid homeostasis via an AMPK-dependent signaling pathway. Targeting AMPK activation by anthocyanin may represent a promising approach for the prevention and treatment of obesity-related nonalcoholic fatty liver disease.

## Background

Nonalcoholic fatty liver disease (NAFLD) is a serious consequence of obesity, increasing the risk of liver cancer or cirrhosis [1]. The origin of this disease is unknown and probably multifactorial. Nevertheless, because impaired lipid metabolism is recognized as an associate and/or promoting mediator of the disease, management of hepatic metabolic disorders becomes an essential strategy for prevention and treatment of obesity-related NAFLD [2].

AMP-activated protein kinase (AMPK) is a key sensor of cellular energy status and it is also recognized as a major regulator of liver and whole body lipid homeostasis [3]. AMPK activation in the liver results in the phosphorylation and inactivation of acetyl-CoA carboxylase (ACC), a direct AMPK substrate, leading to decreased conversion of acetyl-CoA to malonyl CoA [4]. AMPK activation also results in phosphorylation and activation of malonyl CoA decarboxylase (MCD), resulting in further lowering of malonyl CoA levels. Malonyl CoA allosterically inhibits carnitine palmitoyl-CoA transferase 1 (CPT-1), the enzyme responsible for transport of long chain acyl-CoAs into mitochondria for oxidation. Additionally, as malonyl CoA is required for de novo synthesis of fatty acids, decreased malonyl CoA leads to a reduction in hepatic fatty acid synthesis [5]. Therefore, AMPK activation leads to a concomitant inhibition of fatty acid synthesis and activation of fatty acid oxidation.

Anthocyanins are naturally occurring polyphenolic compounds in the plant foods and widely distributed in fruits, vegetables, and pigmented cereals, suggesting that we can ingest significant amounts of anthocyanins from plant-based daily diets [6]. Clinical investigations have indicated that the moderate consumption of anthocyanins through the intake of lyophilized grape powder or berry-derived anthocyanin supplements is associated with improved lipid profile [7,8]. In previous animal studies from us and other investigators, anthocyanins have been shown to ameliorate dyslipidemia and hepatic steatosis in different rodent models [9-12]. However, the molecular mechanism under this action remains unknown and needs further investigation in cells.

Considering the key role of AMPK activation in regulating lipid metabolism and the potential capability of anthocyanin in preventing hepatic steatosis, we observed the influence of cyanidin-3-*O*-β-glucoside (Cy-3-g), the most abundant anthocyanin in plants [6], on AMPK activation and fatty acid metabolism in human HepG2 hepatocytes. The signal pathway of the action involved in AMPK molecular activation was also investigated.

## Results

### Anthocyanin induces AMPK activation in HepG2 cells

**Figure 1:**
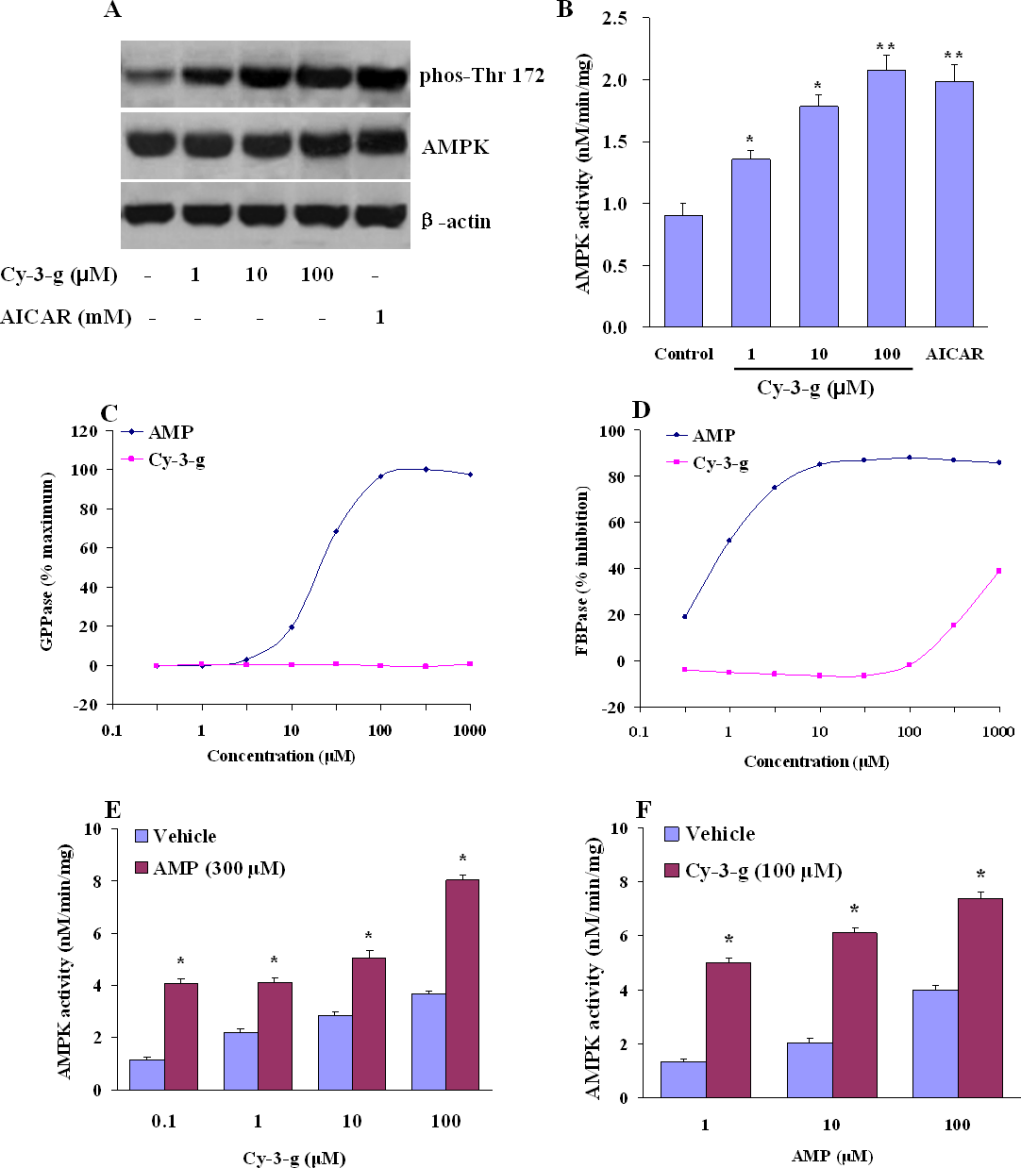
**Anthocyanin activates AMPK in cultured HepG2 cells**. (A) HepG2 cells were treated with various concentrations of Cy-3-g or AICAR (1 mM) for 1 h. The cells were then lysed, and 100 μg of the cell lysates underwent SDS-PAGE followed by Western blot analysis for AMPK phosphorylation. (B) Two microgram of protein extracts were subjected to AMPK activity assays with SAMS peptide as substrates, results are expressed as nmoles ATP/mg protein/min. *P <0.05 or **P <0.01 compared to control. (C) GPPase activity was measured in the presence of glycogen using a phosphoglucomutase and glucose-6 phosphate dehydrogenase-coupled spectrophotometric method. Effects of anthocyanin are expressed as a percent of the maximum stimulation achieved by AMP. (D) FBPase activity was determined using the substrate D-fructose-1,6-bisphosphate and colorimetric detection of free phosphate. Results are expressed as percent inhibition relative to vehicle control. (E) The concentration-dependent AMPK activation by anthocyanin and additive effects in the presence of 300 μM AMP. *P <0.05 compared to vehicle. (F) AMP dose-responsive AMPK activation and additive effects in the presence of 100 μM Cy-3-g. *P <0.05 compared to vehicle. The results presented in panels (B) through (F) are means ± SE from experiments run in triplicate and are representative of at least three independent experiments.

To assess the impact of anthocyanin on AMPK activation, the human HepG2 cells were treated with Cy-3-g of 1~100 μM or with an AMPK activator-AICAR (1 mM) for 1 h, respectively. We first determined the Thr-172 phosphorylation of AMPK because this site is an essential marker of AMPK activity. Cy-3-g significantly stimulated AMPK phosphorylation in a concentration-dependent fashion over the untreated control (Figure 1A). Importantly, the effect of Cy-3-g at a concentration of 10 μM was comparable with AICAR. However, no change in the expression of endogenous total AMPK protein was noted. To further confirm that the effect of anthocyanin on AMPK, the kinase activity of AMPK was assayed by using SAMS peptide as the substrate. Consistent with the increased AMPK phosphorylation, Cy-3-g strongly stimulates AMPK kinase activity in a concentration-dependent manner in HepG2 cells (Figure 1B).

The enzymes fructose1,6-diphosphatase (FBPase) and glycogen phosphorylase (GPPase) are allosterically modulated by AMP. AMP activates GPPase, whereas it inhibits FBPase [13]. To assess the specificity of anthocyanin for AMPK activation, we determined their effects on these two enzymes. Anthocyanin treatment had no effect on GPPase activity at concentrations up to 1 mM, while the EC_50 _of AMP for the activation of GPPase was 18.5 μM (Figure 1C). Similarly, anthocyanin showed no significant inhibitory effect on FBPase at concentrations up to 1 mM, while AMP significantly inhibited FBPase (IC_50 _= 1.6 μM, Figure 1D). These results strongly indicate that anthocyanin stimulates AMPK activity in a manner that differs from AMP. To further address this issue, we evaluated the additivity of AMP and anthocyanin mediated AMPK stimulation. Anthocyanin Cy-3-g increased AMPK activity in the presence of a saturating concentration of AMP (Figure 1E) and AMP stimulated AMPK in the presence of a maximally efficacious concentration of Cy-3-g (Figure 1F). This further supports the hypothesis that anthocyanin binds at a unique site that differs from that of AMP binding.

### Intracellular Ca^2+ ^and CaMKK-β mediates the anthocyanin-induced activation of AMPK

**Figure 2:**
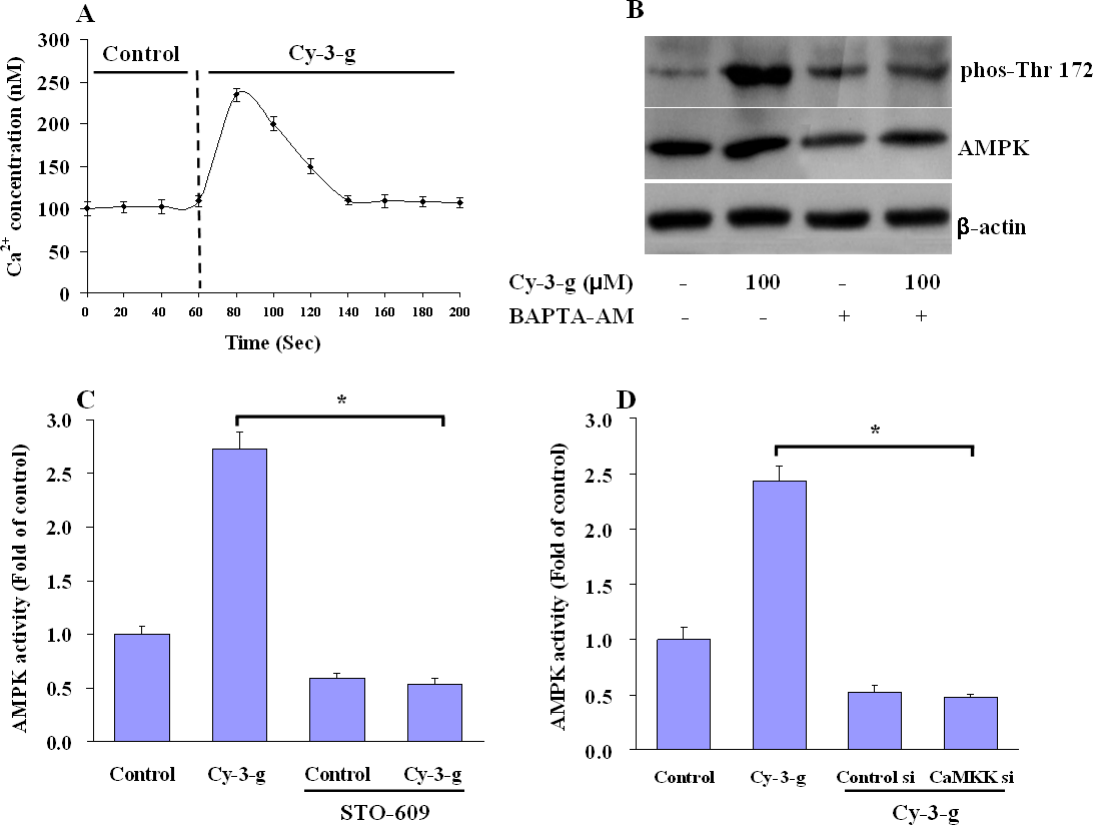
**Anthocyanin-induced activation of AMPK is mediated by Ca^2+ ^and CaMKK-β**. (A) A representative trace of Ca^2+ ^influx into HepG2 cells exposed to anthocyanin is illustrated. Fura-2-loaded cells were initially incubated with media and then switched to anthocyanin. Changes in [Ca^2+^]_i _are expressed as the nM. *n *= 3. (B) HepG2 cells were exposed to media or Cy-3-g for 30 min in the presence or absence of BAPTA-AM (20 μM, 20-min preincubation). AMPK phosphorylation and total AMPK were determined by Western blot. Representative Western blot of pAMPK and total AMPK are shown. (C) HepG2 cells were exposed to culture medium (control) or Cy-3-g (100 μM) for 30 min in the presence or absence of STO-609 (20 μg/mL, 30-min preincubation). AMPK activity was determined as indicated. Values are expressed as fold of control. *n *= 4. *P <0.05 between STO-609 and STO-60 plus Cy-3-g. (D) HepG2 cells were transfected with CaMKK-β siRNA (CaMKK-β si) or scrambled control siRNA (Control si), and 48 h later cells were exposed to medium or Cy-3-g (100 μM) for 30 min. Values are expressed as fold of control. *n *= 4. *P <0.05 between Cy-3-g and CaMKK siRNA plus Cy-3-g.

AMPK is also activated by calmodulin-dependent protein kinase kinase β (CaMKK-β) triggered by a rise in intracellular calcium ions ([Ca^2+^]_i_), without detectable changes in the AMP/ATP ratio [14]. We next investigated whether the Ca^2+ ^signaling was involved in the AMPK activation upon exposure of HepG2 cells to anthocyanin. Anthocyanin exposure resulted in a rapid and transient increase in [Ca^2+^]_i _concentration (Figure 2A). Chelation of intracellular Ca^2+ ^with BAPTA-AM prevented the anthocyanin-induced phosphorylation of AMPK (Figure 2B), suggesting that intracellular Ca^2+ ^was involved in the signaling cascade initiated by the anthocyanin. Furthermore, preincubation of HepG2 cells with STO-609, a CaMKK-β inhibitor, decreased baseline of AMPK activity, and anthocyanin did not increase AMPK activation (Figure 2C), suggesting a role for CaMKK-β. To confirm that CaMKK-β was the kinase upstream of AMPK, a siRNA approach was utilized to knock down CaMKK-β expression in HepG2 cells. Similar to the effects of STO-609, baseline AMPK activity was reduced and remained unchanged upon exposure to anthocyanin (Figure 2D).

### Anthocyanin suppresses ACC activity, decreases malonyl CoA contents and stimulates CPT-1 expression in HepG2 cells

**Figure 3:**
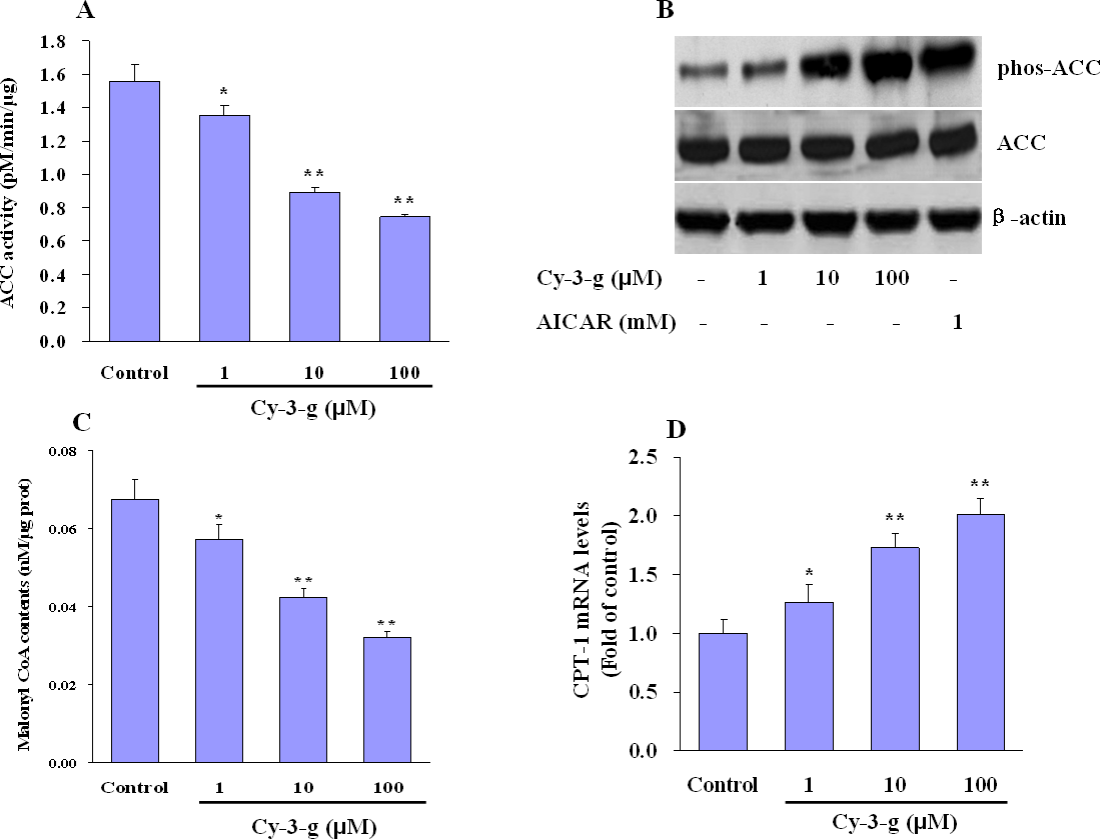
**Anthocyanin suppresses ACC activity, decrease Malonyl CoA levels and prevents FAS expression in HepG2 cells**. (A) Western blot analysis of the phosphorylated form of ACC or total ACC in cell lysates from HepG2 cells treated for 1 h with Cy-3-g or AICAR. (B) ACC activity in cellular extracts was measured and expressed as means ± SE. (C-D) HepG2 cells were left alone or treated with various concentrations of Cy-3-g for 6 h. Then cells were collected and lysed for measuring (C) malonyl CoA levels and (D) CPT1 mRNA expression as indicated. Results are expressed as means ± SE. *P <0.05; **P < 0.01 compared to control.

To determine whether AMPK activation affects its downstream target proteins, we examined the activity of ACC, a key enzyme in the regulation of fatty acid metabolism [5]. Anthocyanin suppressed ACC activity in a dose-dependent fashion in HepG2 cells (Figure 3A). These effects are probably caused by phosphorylation of ACC by AMPK, as ACC serine-79 phosphorylation was increased after anthocyanin treatment, whereas overall ACC protein expression did not change (Figure 3B). AMPK activation and ACC inhibition may lead to a reduction in malonyl CoA levels and release of CPT-1 from allosteric inhibition [15,16]. We next investigated the net effect of these changes in malonyl CoA. Our results show that malonyl CoA levels decreased acutely after anthocyanin treatment in a dose-dependent fashion (Figure 3C), accompanying with a statistically significant and dose-responsive increase of CPT-1 mRNA expression in HepG2 cells (Figure 3D).

### Anthocyanin increases fatty acid oxidation and inhibits lipogenesis in HepG2 cells

**Figure 4:**
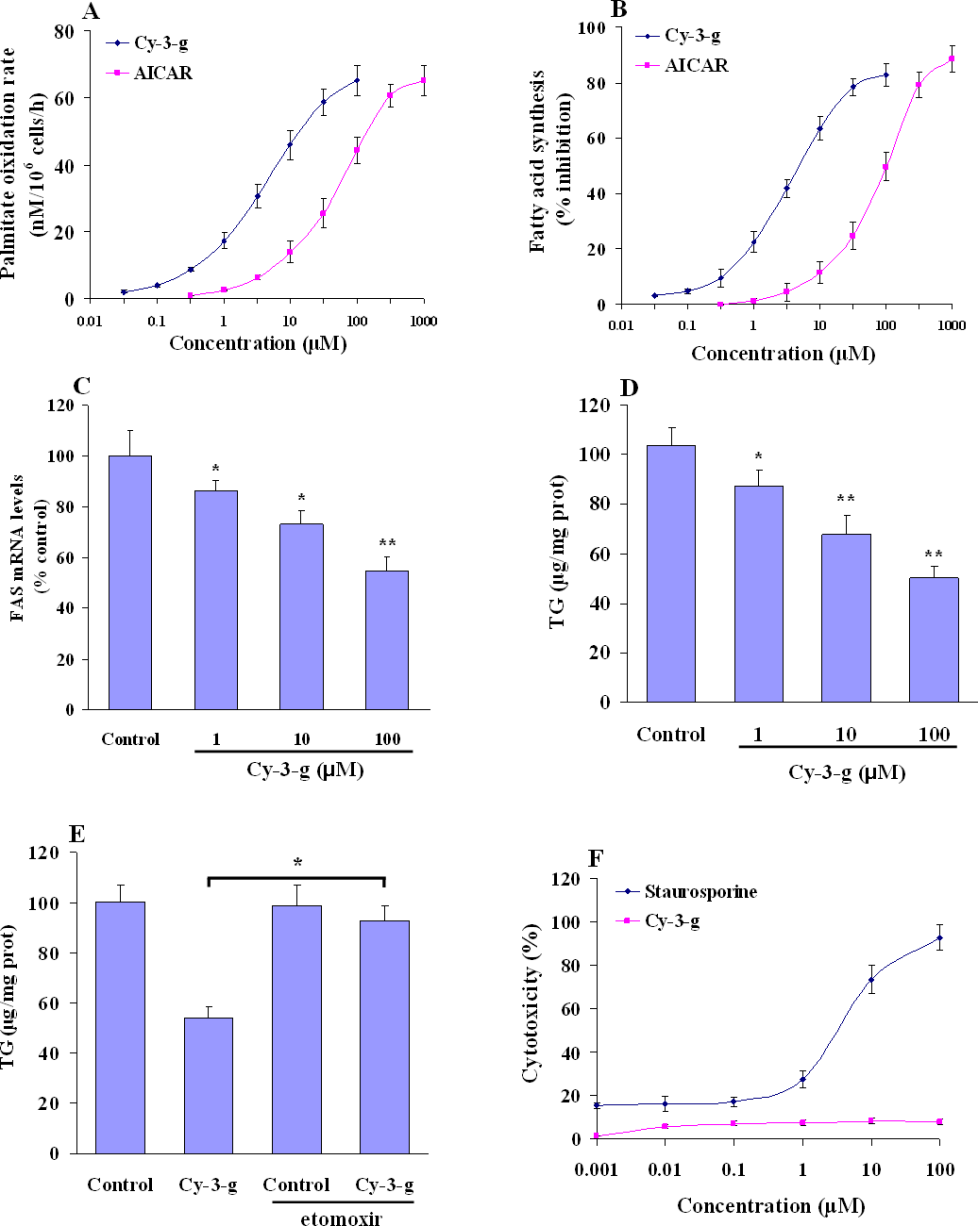
**Anthocyanin increases fatty acid oxidation in HepG2 cells**. (A) Fatty acid oxidation rate was determined by cotreatment with various concentrations of Cy-3-g in the presence of [^14^C]palmitate. Results are expressed as fold of control from at least three independent experiments. (B) Fatty acid synthesis was measured as incorporation of ^14^C-acetate into fatty acids in HepG2 cells during a 6-h treatment with increasing concentrations of Cy-3-g or AICAR, respectively. Results are given as percent inhibition of fatty acid synthesis relative to the control and are means ± SE from triplicate analysis. (C) Dose-dependent inhibition of FAS mRNA expression by Cy-3-g. The results are expressed as percent of control from experiments run in triplicate of three independent experiments. (D) Intracellular TG contents were measured in cell lysates by spectrophotometic methods. Results are means ± SE from triplicate measurements. *P <0.05; **P < 0.01 compared with control. (E) HepG2 cells pre-incubated with vehicle or etomoxir (50 mM) for 1 h were treated with either vehicle (control) or Cy-3-g (100 μM). Then, Intracellular triglyceride contents were determined. *P <0.05 between Cy-3-g and Cy-3-g plus etomoxir. (F) The cellular cytotoxity assays run in HepG2 cells using MTS reagents comparing a known cytotoxin-staurosporine and Cy-3-g. Data are from a minimum of 4 experiments run in triplicate and is demonstrated as means ± SE.

Since AMPK activation inhibits ACC activity, further leading to reduction of malonyl CoA and promotion of CPT-1 activation, one expected consequence is the increased fatty acid oxidation and reduced lipogenesis. To evaluate this hypothesis we studied the effects of anthocyanin on hepatic fatty acid oxidation and synthesis. Anthocyanin functionally enhanced fatty acid oxidation as Cy-3-g increased palmitate oxidation rates in HepG2 cells, with EC_50 _= 5.7 μM for Cy-3-g (Figure 4A). In addition, anthocyanin treatment significantly inhibited the fatty acid synthesis as measured by incorporation of [^14^C]acetate into fatty acids, with IC_50 _= 6.3 μM for Cy-3-g (Figure 4B). The effect of anthocyanin on lipogenesis may be attributed to reduced fatty acid synthase (FAS) gene expression, as Cy-3-g treatment caused a dose-dependent inhibition of FAS mRNA expression in HepG2 cells (Figure 4C). Further measurement showed that Cy-3-g treatment caused a dose-dependent reduction of triglyceride (TG) content in HepG2 cells (Figure 4D). Inhibition of mitochondrial fatty acid oxidation with etomoxir was enough to hamper the decrease in triglyceride levels induced by anthocyanin (Figure 4E), indicating that AMPK-mediated mitochondrial β-oxidation attributed for the major effects of anthocyanin. To exclude the possibility that inhibition of fatty acid metabolism could be mediated in part by cytotoxicity, we assessed the potential cellular cytotoxicity of anthocyanin. Treatment of human HepG2 cells with Cy-3-g at the concentrations up to 100 μM showed no measurable cytotoxicity, whereas staurosporine, a known cytotoxic agent, significantly increased cellular cytotoxicity (Figure 4F). We further probed the mechanism of action by assessing whether anthocyanin alters the cellular levels of AMP and ATP. Cy-3-g treatment at concentrations up to 100 μM caused no significant alterations in ATP and AMP levels in HepG2 cells (Table 1). Taken together, these results indicate the anthocyanin exert their cellular effects by direct activation of AMPK activity and not as a consequence of cellular toxicity, or by altering the AMP/ATP ratio.

**Table 1:** Nucleotide levels following treatment of HepG2 with Cy-3-g

Cy-3-g concentration	AMP (nM/10^6 ^cells)	ATP (nM/10^6 ^cells)	AMP/ATP ratio
Untreated control	0.348 ± 0.021	6.236 ± 0.160	0.056 ± 0.003
1 μM	0.336 ± 0.044	6.525 ± 0.129	0.051 ± 0.007
10 μM	0.398 ± 0.018	6.271 ± 0.087	0.064 ± 0.002
100 μM	0.354 ± 0.011	6.633 ± 0.310	0.054 ± 0.003
1 mM	0.347 ± 0.021	6.412 ± 0.235	0.054 ± 0.006

### AMPK signaling mediates anthocyanin-induced reduction of lipid levels in HepG2 cells

**Figure 5:**
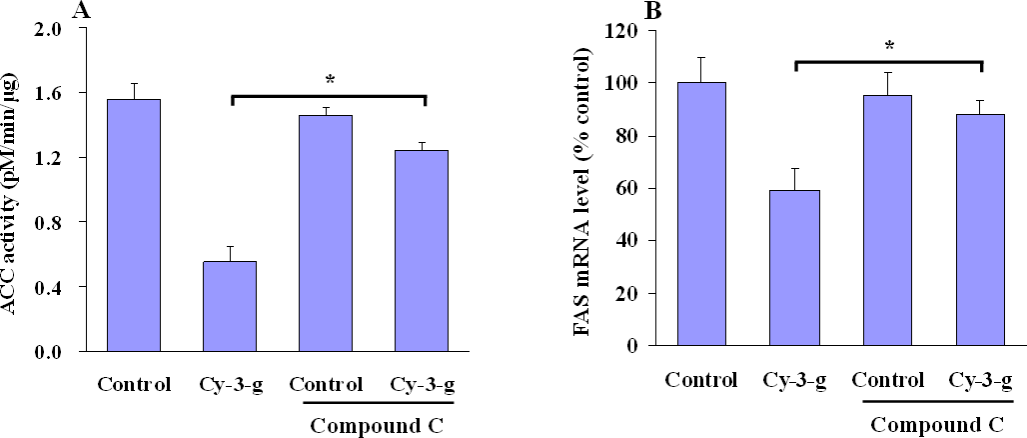
**AMPK inhibition abrogates anthocyanin-mediated increase of ACC phosphorylation and decrease of FAS expression**. HepG2 cells were treated with Cy-3-g (100 μM) in the absence or presence of AMPK inhibitor compound C (10 mM). (A) ACC activity and (B) FAS mRNA expression were analyzed as indicated. Results are expressed as means ± SE. *P <0.05 between Cy-3-g and Cy-3-g plus compound C.

**Figure 6:**
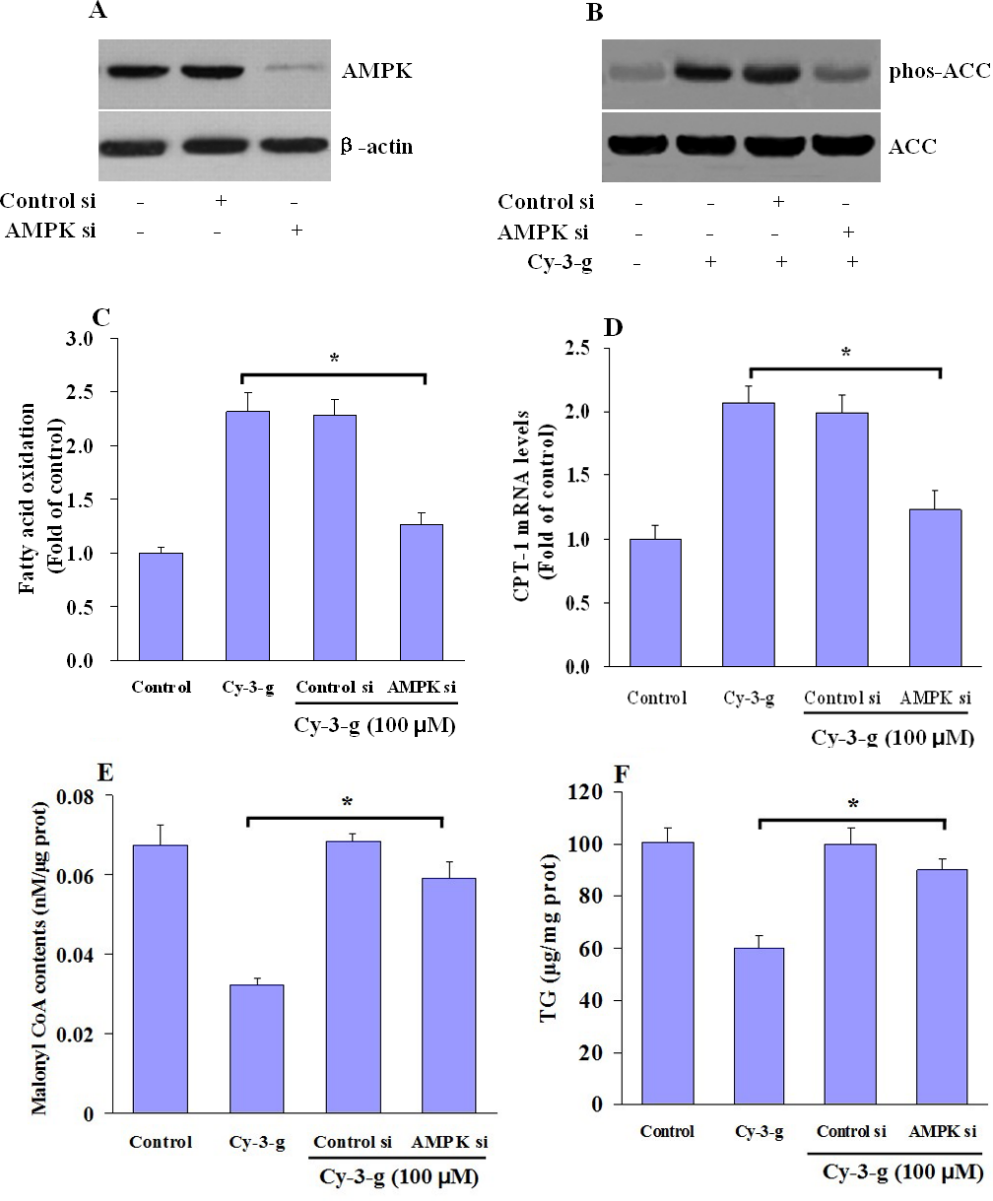
**AMPK mediates anthocyanin-induced lipid reduction in HepG2 cells**. (A) HepG2 cell were transfected with AMPK siRNA. Control siRNA was used as control. AMPK protein expression was determined by Western blot analysis. Representative blots from three independent assays were shown. (B-E) HepG2 cells were transfected with either control siRNA (Control si) or AMPK specific siRNA (AMPK si), followed by treatment with Cy-3-g (100 μM). After then, (B) ACC phosphorylation, (C) fatty acid oxidation, (D) CPT1 mRNA expression, (E) malonyl CoA and (F) hepatic triglyceride were measured and results are shown as means ± SE from triplicate measurements from at least three independent experiments. *P < 0.05 between Cy-3-g and Cy-3-g plus AMPK siRNA.

To elucidate the AMPK pathway involved in regulating hepatic fatty acid metabolism, we applied several independent approaches to inhibit AMPK activation. Pretreatment of HepG2 cells with compound C, a potent and nonspecific inhibitor of AMPK [17], significantly prevented anthocyanin-mediated inhibition of ACC activity (Figure 5A) and FAS expression (Figure 5B) in HepG2 cells. To examine the critical role of AMPK in anthocyanin-mediated hepatic fatty oxidation, we used the specific siRNA against AMPK. Expression of endogenous AMPK protein was remarkably suppressed by AMPK siRNA (Figure 6A), suggesting that the knockdown effect is specific. Compared to control siRNA, AMPK siRNA transfection reversed the phosphorylation of ACC induced by Cy-3-g (Figure 6B), indicating that ACC activity is mainly dependent on the presence of AMPK. In line with this, direct measurement of palmitate oxidation (Figure 6C) confirmed that the effects of anthocyanin on CPT-1 expression (Figure 6D) and malonyl CoA levels (Figure 6E) were blunted in HepG2 cells where AMPK expression was knocked down. The lack of AMPK hence alters the long-term actions of anthocyanin on lipid metabolism (Figure 6F).

**Figure 7:**
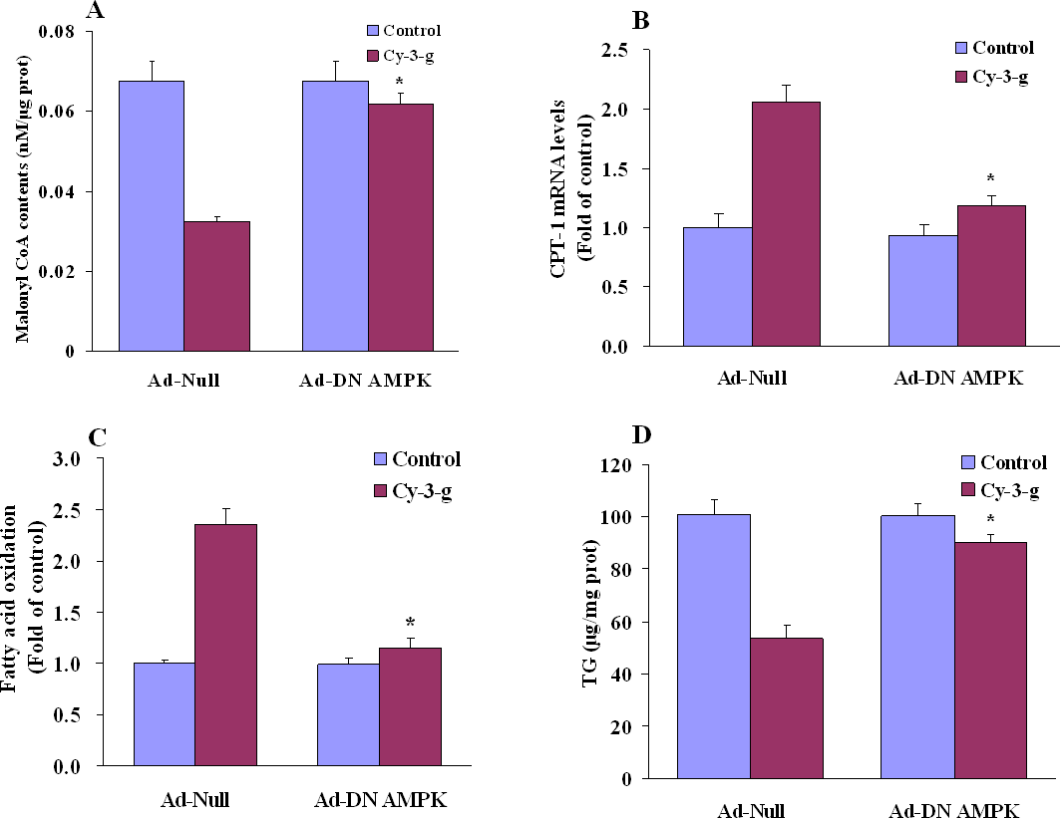
**AMPK depletion abolishes the effects of anthocyanin on hepatic fatty acid metabolism**. HepG2 cells were infected with a control null adenovirus (Ad-Null) or dominant-negative AMPK (Ad-DN AMPK) either with anthocyanin (Cy-3-g, 100 μM) or without anthocyanin (Control). (A) malonyl CoA levels, (B) CPT-1 mRNA expression, (C) fatty acid oxidation, and (D) TG levels were determined as indicated, respectively. Results acquired from three independent experiments are expressed as means ± SE. *P < 0.05 compared to Ad-Null control.

To further rule out that AMPK could participate to sustain the action of anthocyanin, adenoviral overexpression of dominant-negative AMPK mutants (Ad-DN-AMPK) was infected into HepG2 cells. The lack of AMPK significantly compromised the anthocyanin-mediated reduction of malonyl CoA levels (Figure 7A). As a consequence, the DN-AMPK completely blunted the effect of anthocyanin to increase CPT-1 mRNA expression (Figure 7B) and fatty acid oxidation (Figure 7C), reversing the inhibition of triglyceride accumulation by anthocyanin (Figure 7D). Together, these data indicate that, to a large extent, AMPK functions as a novel downstream effector in regulation of lipid metabolism by anthocyanin.

## Discussion

This study demonstrates that Cy-3-g, a typical anthocyanin pigment, stimulates AMPK activation via CaMKK in HepG2 cells. The increase in AMPK activation by anthocyanin suppresses ACC activity, causes the reduction in malonyl CoA levels and stimulation of CPT-1, leading to enhanced fatty acid β-oxidation and finally inhibiting the lipid accumulation in HepG2 cells. Thus, the novel findings from the present study is that anthocyanin beneficially regulates hepatic fatty acid metabolism through AMPK-ACC-malonyl CoA-CPT1 pathway.

Previous studies had shown that Cy-3-g activated AMPK and enhanced adipocytokine secretion and adipocyte-specific gene expression in isolated adipocytes [18,19]. Recently, Takikawa et al. have demonstrated that anthocyanin-rich bilberry extract effectively ameliorates hyperglycemia and insulin sensitivity via activation of AMPK in diabetic mice [20]. The results of these studies suggest that anthocyanin may be a potential AMPK activator. However, the impact of anthocyanin on hepatic AMPK and its-mediated physiological consequences on fatty acid metabolism is still undefined. In this study, we identified that anthocyanin Cy-3-g is a potent activator of AMPK in hepatocytes. Meanwhile, GPPase and FBPase activity were not altered in response to anthocyanin, implying that anthocyanin activated AMPK via a unique pathway unlike AICAR/AMP.

In mammals, LKB1 and CaMKK-β have been identified as upstream kinases of AMPK, since both kinases can phosphorylate Thr172 and thus activate AMPK [14,21]. The phosphorylation of Thr172 by LKB1 requires an increase in AMP, while phosphorylation of Thr172 by CaMKK-β is independent of AMP levels and is triggered by a rise in intracellular Ca^2+^. We found that exposing HepG2 cells to anthocyanin initiated a rapid and transient influx of Ca^2+ ^into the cells and that AMPK activation by anthocyanin was prevented by the intracellular Ca^2+^-chelating agent, BAPTA-AM, suggesting that the anthocyanin-induced signaling pathway was dependent upon intracellular Ca^2+^. Since CaMKK-β-mediated stimulation of AMPK required an increase in intracellular Ca^2+ ^levels, we hypothesized that CaMKK-β may be the upstream kinase induced by anthocyanin. Indeed, the CaMKK inhibitor STO-609 decreased the baseline AMPK activity and prevented activation of the AMPK upon anthocyanin exposure. Moreover, when transfected with CaMKK-β siRNA, both baseline and anthocyanin-induced activity of AMPK were inhibited. These data suggest that the anthocyanin-induced activation of AMPK was mediated by CaMKK-β independently of LKB1 signaling pathway.

In the liver, AMPK coordinates the changes in the activity of enzymes of lipid metabolism and regulates the partitioning of fatty acid both in oxidative and biosynthetic pathways [22]. In accordance with this, we observed that anthocyanin treatment induced ACC phosphorylation, depressed its enzymetic activity in HepG2 cells. Inhibition of ACC by AMPK leads to a fall in malonyl CoA content and a subsequent decrease in fatty acid synthesis and increase in mitochondrial fatty acid oxidation via the allosteric regulation of CPT-1, which catalyses the entry of long-chain fatty acyl-CoA into mitochondria [14]. Our results show that malonyl CoA is robustly decreased after anthocyanin treatment, as a result of AMPK and ACC phosphorylation. This resulted in a decrease in malonyl CoA levels associated with increased CPT-1 expression, which resulted in increased fatty acid oxidation. The ability of anthocyanin to stimulate AMPK and reduce hepatocellular lipid accumulation was abolished by pharmacological inhibition or knockdown of AMPK. Thus, anthocyanin-dependent AMPK activation functionally inhibits ACC and FAS which are two key downstream regulators of AMPK in the control of lipid metabolism and accumulation, possibly through increased fatty acid oxidation.

## Conclusion

In summary, our present study provide the mechanistic data that activation of hepatic AMPK by anthocyanin acts upstream on lipid metabolism by activating the pathways controlling the oxidation of fatty acids. Although it appears hard to get the concentration of anthocyanin shown in this study from daily consumed foods, our findings provide a novel insight into the therapeutic implications of anthocyanin in obesity-related liver disorders.

## Methods

### Reagents

Anthocyanin cyanidin-3-*O*-β-glucoside (Cy-3-g; HPLC grade) was provided by Polyphenol AS (Sandnes, Norway). Dulbecco's modified Eagle's medium (DMEM), fetal bovine serum (FBS), and antibiotic mixture (penicillins-treptomycin) were purchased from the Gibco BRL (Grand Island, NY). 5-aminoimidazole-4-carboxamide 1-ribofuranoside (AICAR), anti-phospho-AMPK Thr-172, anti-phospho-ACC Ser-79 and anti-β-actin were purchased from Cell Signaling Technology, Inc. (Danvers, MA). AMPK inhibitor compound C and calmodulin-dependent kinase kinase (CaMKK) inhibitor STO-609 were obtained from Calbiochem (San Diego, CA). The specific AMPK-targeted SAMS peptide used in AMPK activity assay was purchased from GenScript (Piscataway, NJ); siRNA against CaMKK-β was from Qiagen, Inc. (Valencia, CA); and all other chemicals, unless otherwise specified, from Sigma-Aldrich (St. Louis, MO).

### Cell culture

HepG2 cells (human hepatoma cell line, ATCC #HB 8065, American Type Culture Collection) were cultured in DMEM supplemented with 10% FBS and antibiotics at 37°C in a humidified, 5% CO_2_/95% air atmosphere. After reaching ~80% confluence, cells were serum-starved for 24 h in medium containing 0.5% FBS.

### Adenoviruses

The adenoviral vector expressing a dominant-negative AMPKα mutant (Ad-DN-AMPK) was kindly donated by Dr J. Ha (Department of Molecular Biology, Kyung Hee University, College of Medicine, Seoul, Korea).

### AMPK activity

AMPK activity was measured by monitoring phosphorylation of the SAMS peptide substrate as previously described [13]. To determine whether compound-induced AMPK activation occurs in a reversible manner, AMP or Cy-3-g were preincubated with rat liver AMPK for 10 min at 20 times standard assay concentrations prior to dilution and measurement of AMPK activity.

### Glycogen phosphorylase assay

The rabbit glycogen phosphorylase (GPPase) at the concentration of 1.5 μg/mL of was added to a reaction mixture containing 20 mM Na_2_HPO_4 _(pH 7.2), 2 mM MgSO_4_, 1 mM β-NADP (β-nicotinamide adenine dinucleotide phosphate), 1.4 U/mL G-6-PDH (glucose-6-phosphate-dehydrogenase) and 3 U/mL PGM (phosphoglucomutase). AMP or Cy-3-g was added to the assay medium at the specified concentrations followed by the addition of glycogen (final concentration 1 mg/mL) to initiate the reaction. After incubating 10 min at 25°C, GPPase activity was assessed by measuring absorbance at 340 nm [23].

### Fructose 1,6-bis-phosphatase assay

D-fructose-1,6 diphosphatase (FBPase, 0.01 U/mL final concentration) in 2× assay buffer, 100 mM Tris (pH 7.0), 4 mM MgCl_2_, 300 mM NaCl, 0.2 mg/mL BSA and 6 mM DTT, was added to test compounds to give final concentrations as indicated. Substrate, D-fructose-1,6-diphosphate, at a final concentration of 0.1 mM was added to the reaction and the reaction mix was incubated at 30°C for 20 min. Following the incubation 2 volumes of Malachite Green solution (Upstate Inc., Waltham, MA) containing 0.001% Tween 20 was added and absorbance at 640 nm read immediately [24].

### Measurement of intracellular ATP and AMP levels

To measure adenine nucleotide concentrations in HepG2 cells, each well of HepG2 cells in 6-well plates was harvested in 300 μL of lysis buffer containing 5% (v/v) perchloric acid. After sonication for 5 s, the samples were centrifuged at 16,000 × g for 10 min at 4°C twice to remove acid-insoluble material. The supernatant neutralized with KOH and analyzed by HPLC (Waters, Milford, MA) with an LC-18T reverse-phase column (Supelco, Bellefonte, PA) at a flow rate of 1 mL/min. Nucleotides were detected by their absorbance at 254 nm and compared with external AMP and ATP standards to confirm its identity [25].

### Live cell intracellular calcium measurements

The fluorescent calcium indicator, fura-2-acetoxymethyl ester (fura-2-AM; Molecular Probes, Eugene, OR), was used to measure changes in intracellular free calcium ([Ca^2+^]_i_). Cells were incubated at 25°C for 60 min with 1 μM fura-2-AM in modified Krebs Ringer buffer [(in mM) 120 NaCl, 5 KCl, 1.2 CaCl_2_, 0.7 MgSO_4_, 15 N-2-hydroxyethylpiperazine-N'-2-ethanesulfonic acid (HEPES), and 1.8 g/L glucose (pH 7.4)]. After dye loading, cells were washed three times in the same medium and kept in the dark for at least 30 min before single-cell [Ca^2+^]_i _measurements. Changes in [Ca^2+^]_i _were determined ratiometrically (340 nm/380 nm excitation, 512 nm emission) in 1-mL aliquots using a spectrofluorometer. Calcium concentrations were calculated using the equation: [Ca^2+^]_i _= K_d_(F_380 max_/F_380 min_) (R - R_min_)/(R_max _- R) [26]. A dissociation constant (K_d_) value of 224 nM was assumed for the binding of calcium to fura-2-AM. R_max _and R_min _were determined in each experimental group by the consecutive addition of 30 μM digitonin (R_max_) and 50 mM EGTA (R_min_). R_max _and R_min _are the maximum and minimum F_340_/F_380 _ratios, respectively. F_380 max_/F_380 min _= the ratio of fluorescence emission intensity at 380-nm excitation in Ca^2+^-depleting (F_380 max_) and Ca^2+^-saturating (F_380 min_) conditions.

### SiRNA transfection

AMPK siRNA SMARTpool™ were purchased from Upstate Biotechnology (Chicago, IL). A nonrelated, scrambled siRNA pool (Dharmacon; Lafayette, CO) was used as a negative control. For AMPK knockdown, HepG2 cells were plated in 6-well plates and transfected with 50 nM siRNA (AMPK SMARTpool or nonspecific siRNA pool) using Oligofectamine reagent (Invitrogen; cat. no. 12252-011) following the manufacturer's instruction. Real-time PCR was used to evaluate AMPK knockdown efficiency after cells were incubated in transfection medium for 48 h.

### Quantitative Real-Time PCR

Total RNAs were extracted from HepG2 cells using TRIzol reagent (Invitrogen) according to the manufacturer's protocol. The reverse-transcription reaction was performed using 1 μg total RNA that was reverse-transcribed into the first-strand cDNA by Superscript II reverse transcriptase with random primers (Invitrogen). The cDNA levels were quantified by real-time PCR with SYBR Green using a Platinum qRT-PCR kit (Invitrogen) on an ABI 7700 sequence detection system (PerkinElmer Biosystems). Primers are shown as follows: human CPT-1 (Accession no. NM_001876), Forward: 5'-CGTCTTTTGGGATCCACGATT-3', Reverse: 5'-TGTGCTGGATGGTGTC TGTCTC-3'; human FAS (Accession no. NM_004104), Forward: 5'-CTGTCTAGGTTTGATG CCTCCT -3', Reverse: 5'-GATCCGAGGGCCTCACTAAAC-3'. Reactions were carried out at least in duplicate for each sample on an ABI 7700 sequence detection system (PerkinElmer Biosystems) according to the manufacturer's instructions, and target values were normalized to glyceraldehyde-3-phosphate dehydrogenase (GAPDH) as indicated. Exponential amplification efficiency was verified during each PCR run using a standard dilution series made from pooled samples. Results were calculated from the difference in threshold cycle values for CPT-1, FAS and GAPDH.

### Quantification malonyl-CoA

For quantification of malonyl-CoA in cells, radiolabeled palmitate stoichiometrically converted from malonyl-CoA in the presence of [^14^C]acetyl-CoA, and purified fatty acid synthetase (FAS) was measured using a modification of a previously described method [27]. The radiolabeled palmitoyl-CoA was extracted with hexane, and radioactivity was measured using a liquid scintillation counter (Canberra-Packard, Zellik, Belgium).

### Measurement of triglyceride content

To assay triglyceride contents in HepG2 cells, 30 μL of triglyceride standard or cleared cell supernatant was added to a 96-well flat bottom polystyrene plate, and 300 μL of infinity triglyceride reagent was then added to the microplate. After cooling to room temperature, samples were read at 520 nm with a Microplate Spectrophotometer (Bio-Tek Instruments Inc., Winooski, VT). Triglyceride levels were normalized to protein concentrations and expressed as μg of lipid/mg of protein.

### Cytotoxicity assay

HepG2 cells (1 × 10^4 ^cells) were plated in 96-well plates and cultured for 12 h in DMEM containing 10% FBS. The cells were then treated with Cy-3-g or staurosporine at the concentrations of 0.001~100 μM for 16 h. The cell viability was determined by WST assay using Cell Proliferation Reagent WST-1 (Roche). Plates were incubated at 37°C to allow for color development and cell viability was evaluated by conversion of a tetrazolium salt into a formazon product measured spectrophotometrically at 490 nm.

### Fatty acid β-oxidation

HepG2 cells treated with vehicle or Cy-3-g were incubated with 500 mM palmitic acid/BSA in maintenance medium for 24 h followed by incubation with 125 mM [^3^H]palmitic acid/BSA and 1 mM carnitine in PBS for 2 h. ^3^H_2_O was measured as described previously [4].

### Fatty acid synthesis assay

HepG2 cells (5 × 10^4 ^per well) were plated on Costar black-walled 96-well assay plate and pretreated as previously described [28]. After 16-hour incubation, the pretreated medium was removed and replaced with medium containing [^14^C]acetate (2 μCi/mL, Perkin Elmer) and AICAR or Cy-3-g at the indicated concentrations. Cells were incubated 4 h at 37°C then the plates were rinsed with PBS. The final wash was replaced with Microscint20 (Perkin Elmer) and radioactivity incorporated into fatty acid monitored on MicroBeta Liquid Scintillation and Luminescence Counters.

### Western blot

Cellular protein extracts were prepared with lysis buffer (50 mM Tris-HCl, pH 8.0, 150 mM NaCl, 0.5% NP40) containing complete protease and phosphatase inhibitors (Roche Applied Science, Penzberg, Germany), and then immunoblotted with antibodies against p-AMPK, AMPK, and p-ACC, ACC for 2 h. Specific antibody binding was detected by horseradish peroxidase-conjugated secondary antibodies and visualized using enhanced chemiluminescence detection reagent (Santa Cruz). The band densities were quantified using an image analyzer Quantity One System (Bio-Rad, Richmond, CA). All protein quantifications were adjusted for the corresponding β-actin level, which was not consistently changed by the different treatment conditions.

### Statistical analysis

Statistical analyses were performed using the SPSS 14.0 package (SPSS Inc., Chicago, IL). All results are expressed as means ± SE. and analyzed by the Student's *t *test or analysis of variance (ANOVA) to determine *P *values; *P *< 0.05 was considered statistically significant.

## Abbreviations

**ACC: **acetyl-CoA carboxylase; **AMPK: **AMP-activated protein kinase; **CaMKK: **calmodulin-dependent protein kinase kinase; **CPT1: **carnitine palmitoyl-CoA transferase 1; **Cy-3-g: **cyanidin-3-*O*-β-glucoside; **FAS: **fatty acid synthase; **FBPase**: fructose-1,6 diphosphatase; **GPPase: **glycogen phosphorylase; **MCD**: malonyl CoA decarboxylase; **NAFLD: **nonalcoholic fatty liver disease; **TG: **triglyceride

## Competing interests

The authors declare that they have no competing interests.

## Authors' contributions

HG and MX conceived the idea and designed the study. DW and GL carried out the lipid metabolism assay experiments. RZ performed the statistical analysis and interpretation of the data. YW participated in the experiments related to AMPK enzyme activity assay. HG drafted the manuscript and MX provided critical corrections to the manuscript. All authors read and approved the final manuscript.
